# Supplementary material for: Neurite Outgrowth and Morphological Changes Induced by 8-trans Unsaturation of Sphingadienine in kCer Molecular Species
Source: Int J Mol Sci. 2019 Apr 29;20(9):2116. doi: 10.3390/ijms20092116 (PMC6540580; doi:10.3390/ijms20092116)

Fig. S1

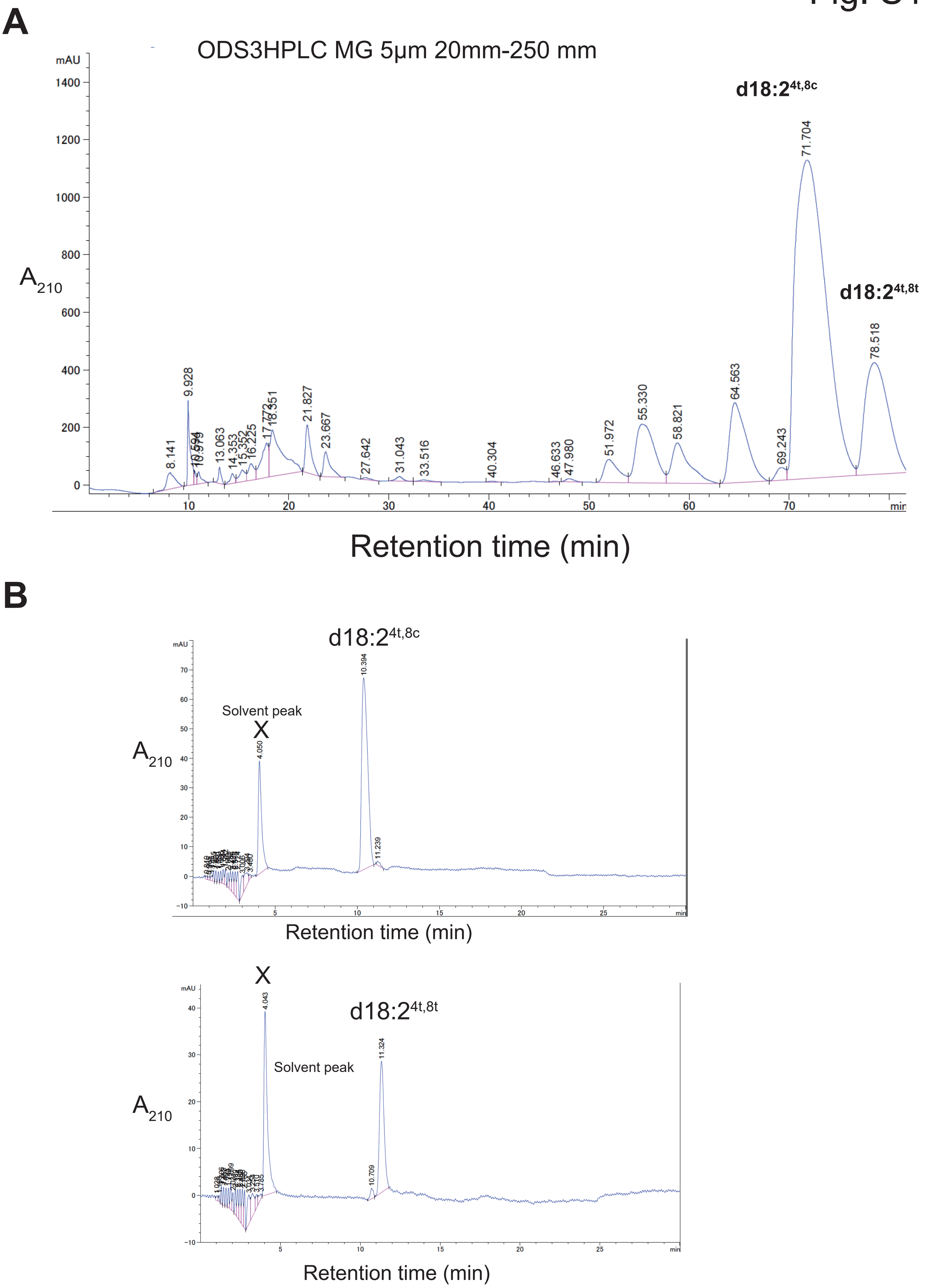
A
ODS3HPLC MG 5µm 20mm-250 mm
mAU
A210
d18:2 4t,8c
d18:2 4t,8t
71.704
78.518
64.563
69.243
55.330
58.821
51.972
46.633
47.980
40.304
33.516
31.043
27.642
23.667
21.827
18.551
17.772
16.225
15.352
14.353
13.063
10.594
10.979
9.928
8.141
Retention time (min)
B
Solvent peak
X
4.050
10.394
11.239
Retention time (min)
4.043
10.709
11.324
Retention time (min)

Fig. S2

A

ODS-HPLC MG 5µm 4.6-250 mm

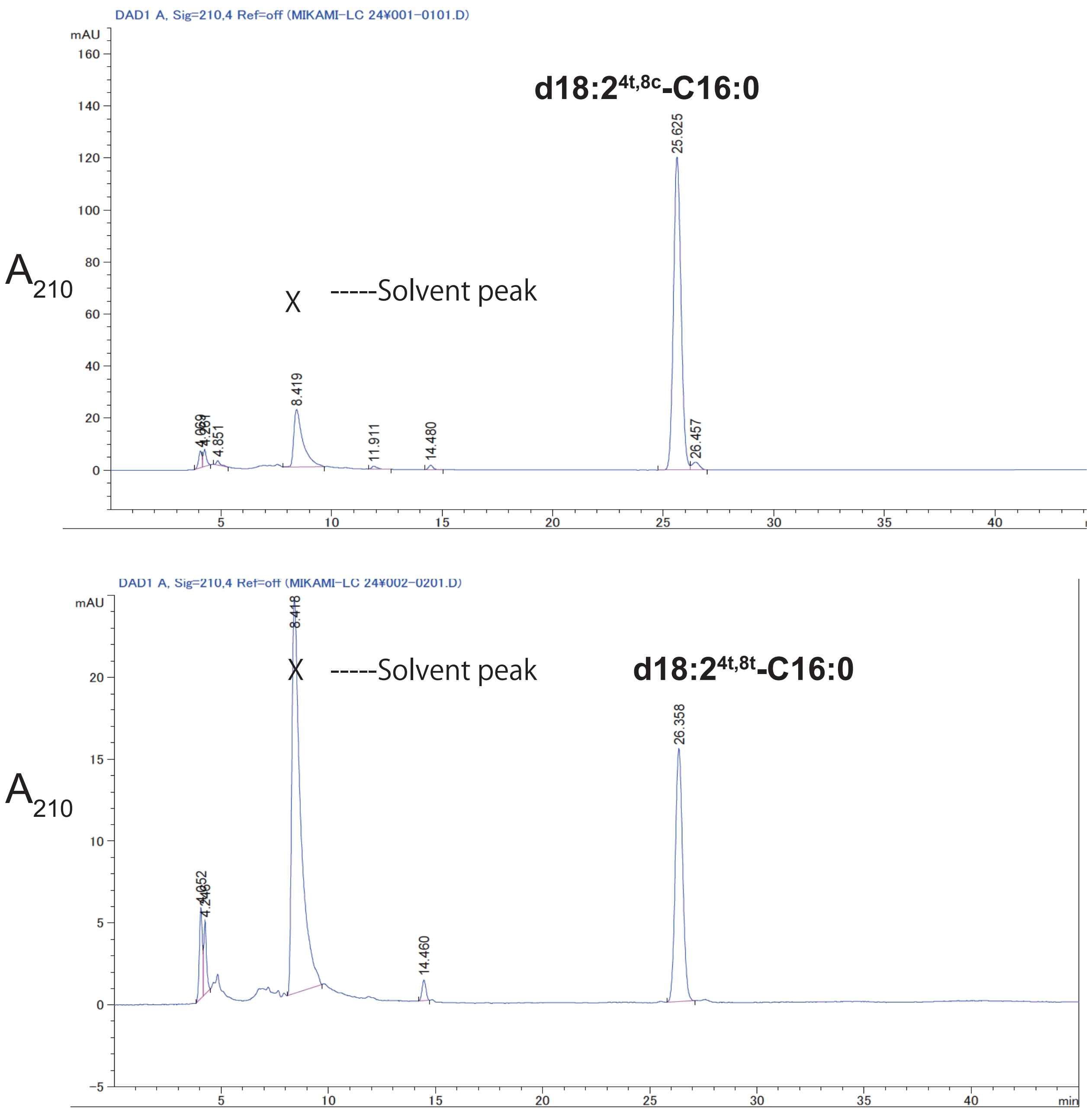

B

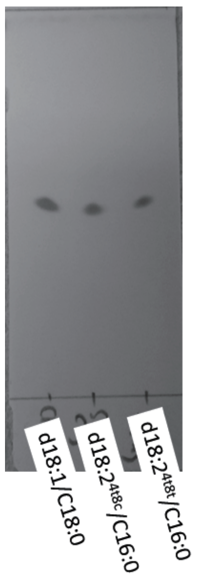

TLC plate developed by CHCl3/MeOH/AcOH (190:9:1, vol/vol/vol) twice/ Visualized with 10 % CuSO4-8% H3PO4 reagent

Fig. S3

A

MS1

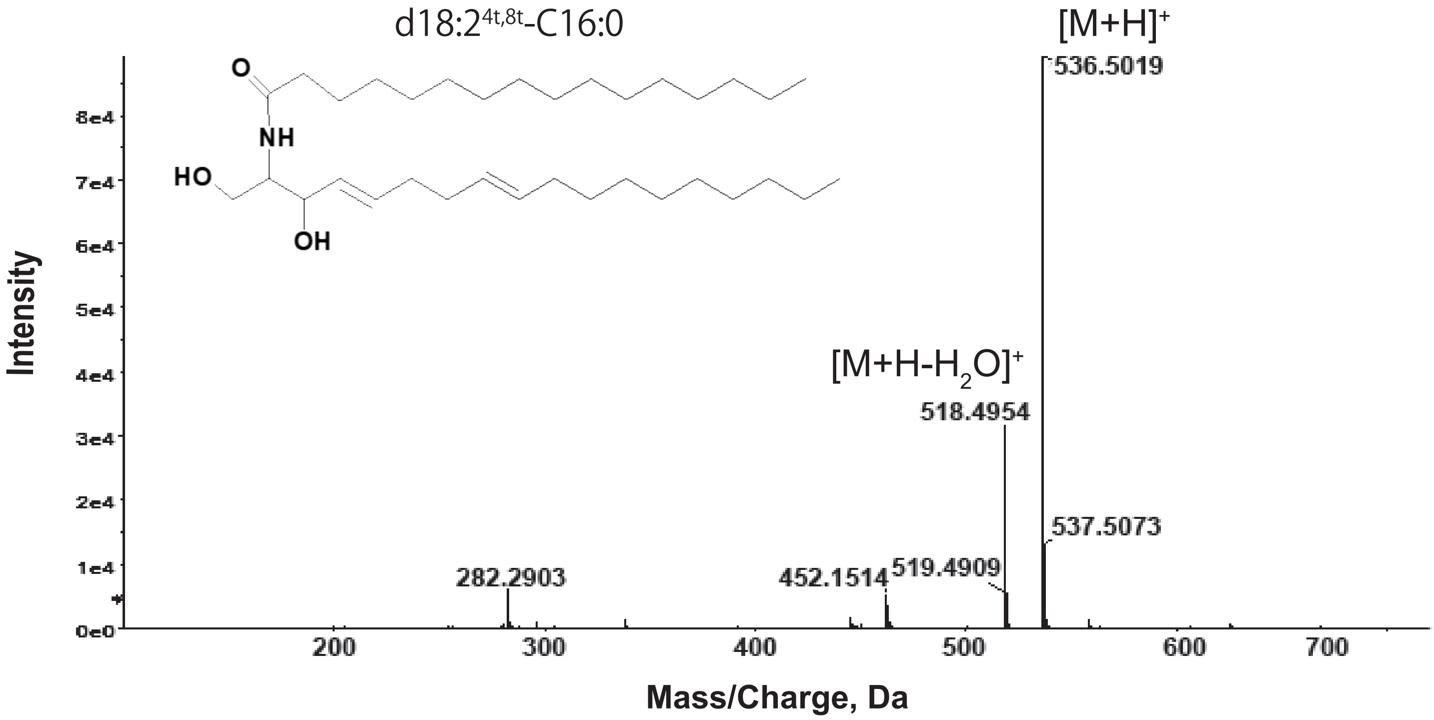

B

MS2

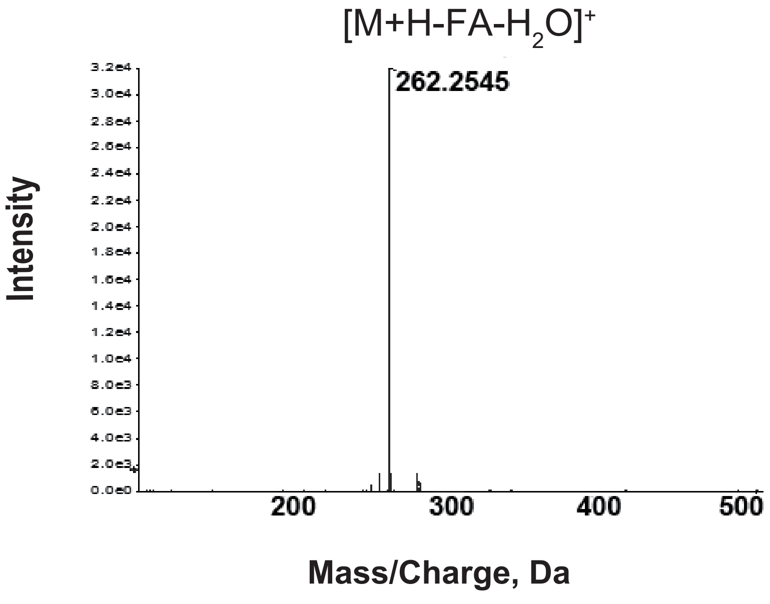

Fig. S4

A

MS1

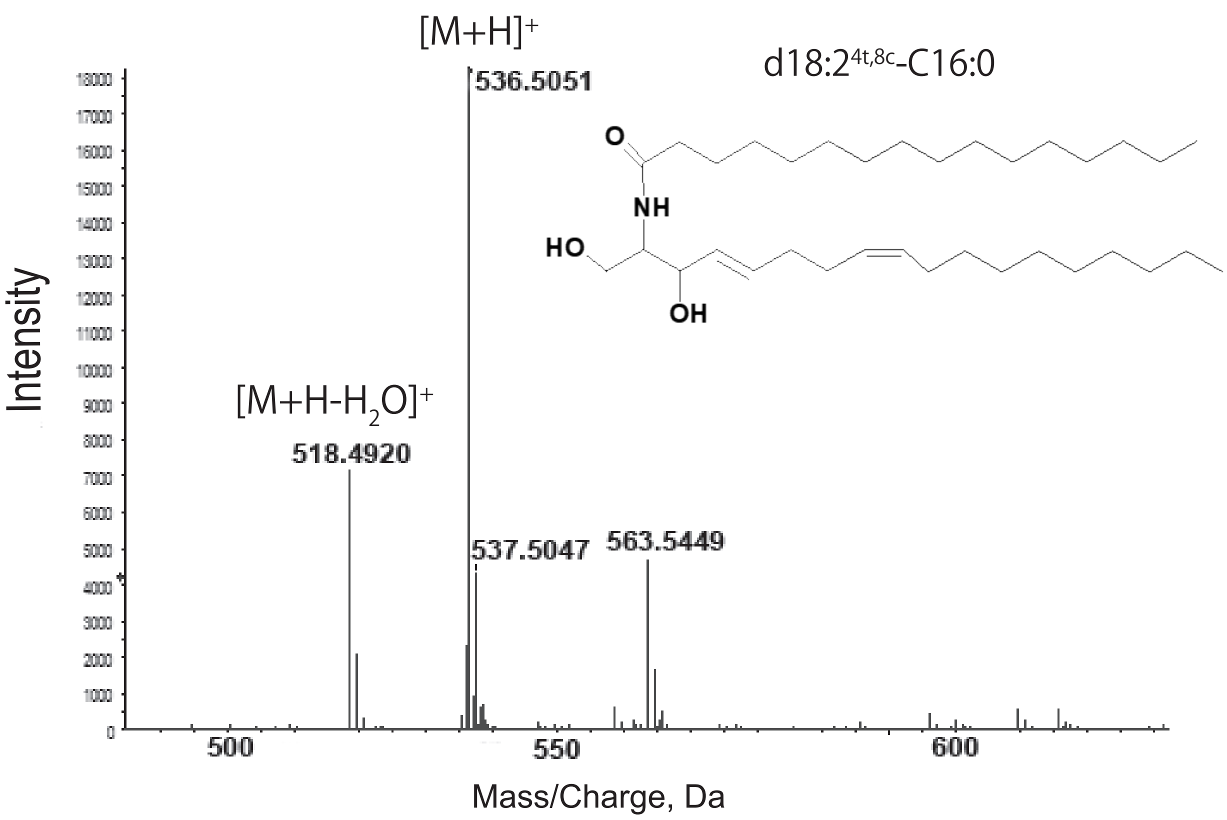

B

MS2

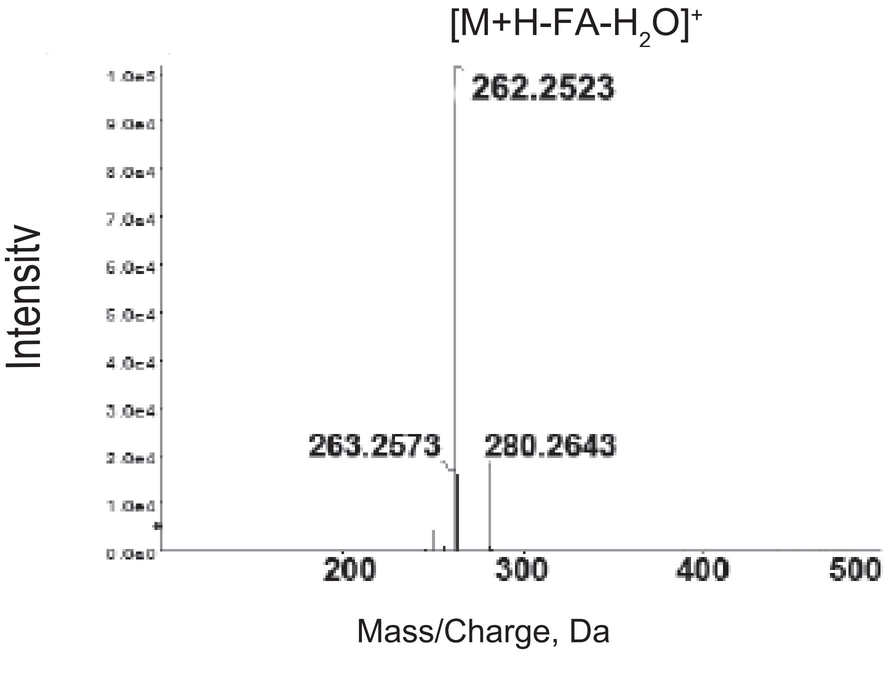

Supplement: Supplementary file 1 [file ijms-20-02116-s001.pdf]
